# Prevalence of *Dermanyssus gallinae* (Mesostigmata: Dermanyssidae) in industrial poultry farms in North-East Tunisia

**DOI:** 10.1051/parasite/2013043

**Published:** 2013-10-28

**Authors:** Mohamed Gharbi, Nadhem Sakly, Mohamed Aziz Darghouth

**Affiliations:** 1 Laboratoire de Parasitologie, Université de la Manouba, École Nationale de Médecine Vétérinaire de Sidi Thabet 2020 Sidi Thabet Tunisia

**Keywords:** *Dermanyssus gallinae*, Poultry, Mite, Tunisia

## Abstract

*Dermanyssus gallinae* (Mesostigmata: Dermanyssidae), a mite of poultry, represents the most important ecotoparasite of egg-laying poultry in several countries. We estimated the prevalence of *D. gallinae* infestation in 38 industrial poultry farms (28 egg-laying and 10 reproductive hen farms) in the governorate of Nabeul (North-East Tunisia). Traps were placed in two locations of each farm during 24 h in August. The overall prevalence at the farms was estimated to be 34%. A total number of 329 *D. gallinae* were collected, giving an intensity of 0.0028 and an abundance of 0.0015. Infestation intensity and abundance were significantly higher in egg production farms than reproductive farms. There was no correlation between the intensity of infestation and temperature. An exponential correlation was observed between the birds’ age and infestation intensity. We recommend a systematic survey of poultry farms during the whole breeding period. Prompt treatment is recommended to avoid the exponential increase of mite population.

## Abstract (fr)

L’acarien de volailles *Dermanyssus gallinae* (Mesostigmata: Dermanyssidae) représente l’ectoparasite le plus important en élevages de poules pondeuses dans plusieurs pays. Nous avons estimé la prévalence de l’infestation dans 38 élevages industriels de volailles (28 élevages de poules pondeuses et 10 élevages de reproducteurs) dans le gouvernorat de Nabeul (nord est de la Tunisie). Les pièges ont été placés à deux endroits de chaque ferme pendant 24 heures au mois d’août. La prévalence dans les élevages a été estimée à 34 %. Un nombre total de 329 *Dermanyssus gallinae* ont été collectés, donnant une intensité d’infestation de 0,0028 et une abondance de 0,0015. L’intensité et l’abondance d’infestation étaient significativement plus élevées dans les élevages de poules pondeuses que les élevages de reproducteurs. Il n’y avait pas de corrélation entre l’intensité d’infestation et la température ambiante. Une corrélation exponentielle a été observée entre l’âge des volailles et l’intensité d’infestation. Nous recommandons une surveillance systématique des élevages industriels de volailles durant toute la période d’élevage. Un traitement rapide est recommandé pour éviter une augmentation de la population des acariens.

## Introduction

*Dermanyssus gallinae* De Geer, 1778 [4] (Mesostigmata: Dermanyssidae) is the most important worldwide-distributed ectoparasite in poultry farming. It infests mainly farms with long production cycles (egg-laying poultry), causing anaemia and pruritus of different intensities, a drop in egg production and transmits several pathogens to the poultry [15]. The prevalence of infestation is high in free range and cage rearing European farms. Indeed, Hamidi et al. [7] reported that 50% of Kosovan free range rearing farms were infected by *D. gallinae*. In cage rearing farms, the prevalence is also high, as estimated in France (56%), the UK (60%) and Denmark (68%) [13]. Moreover, this parasite is zoonotic, inducing serious discomfort to the working staff in affected poultry premises, and is frequently underdiagnosed [2]. The parasites are predominantly hidden in cracks and crevasses during the day, and can survive for several months out of the hosts. The control of this parasite represents a real challenge in egg-laying poultry farms since the use of chemical insecticides is forbidden, but several studies, even in European countries, showed illegal use of acaricides, leading to a high prevalence of laying hen contamination. Indeed, Marangi et al. [9] reported that 37/45 poultry farms were contaminated by carbaryl, an acaricide banned in Europe 3 years ago. Since there are no available specific registered medicines for poultry mite control in Tunisia, farmers use acaricides exclusively during the cleaning time between batches, with unreliable results. To overcome this problem, several recent publications have screened vaccine candidates and evaluated medicinal plants that could offer sustainable and environmentally friendly tools for poultry red mite control [5–7, 11].

As far as could be ascertained from the literature, there is no published report on *D. gallinae* infestation in birds in Tunisia, despite its evident high financial impact in the European Union. Indeed, the total annual costs in the European Union were estimated to be €130 million [12]. This survey aimed to estimate the epidemiological indicators of *D. gallinae* infestation in industrial poultry farms in the governorate of Nabeul (North-East Tunisia).

## Material and methods

The present survey was carried out during August 2011 in 38 randomly included poultry farms in the governorate of Nabeul (North-East Tunisia). This region is located 60 km away from Tunis, with a mean annual rainfall varying between 390 and 630 mm and a mean temperature ranging between 8 °C (from December to March) and 32 °C (July and August).

The governorate of Nabeul has a total of 775 broiler poultry farms, 44 laying hen farms, 18 breeding hen farms, and three hatcheries. We included in the present survey 38 units, consisting of 28 egg-laying and 10 reproductive hen farms.

In laying hen units, birds (Shaver 2000, Lohmann and Babcok 300 breeds) were reared five to a cage in battery cages at a density of 1 hen/550 cm^2^ for 36 to 80 weeks at a room temperature varying between 29 and 32 °C. The reproductive hen units contained birds of both sexes (Shaver 2000, Lohmann and Babcok 300 breeds), aged between 40 and 44 weeks. The birds were kept on the ground at a density of 4 birds/m^2^.

Mites were collected with cardboard traps, which consisted of a 7 × 20 cm piece of cardboard closed on two edges, offering shelter to the mites during the off-host period [7]. The traps were placed for 24 h, out of reach of the chickens, in battery cages for laying hen units and in the nests for reproduction poultry. The traps were collected in plastic bags and stored for 24 h at −20°C, then immerged in isotonic sodium chloride solution. The solution was filtered and the parasites were collected, then counted and identified under a stereomicroscope according to the key of Moss [10].

The unit infestation prevalence (number of infested units/number of visited units), the infestation intensity (number of parasites/number of animals in infested units) and the abundance (number of parasites/number of animals in visited units) were estimated.

Fisher’s exact test was used to compare farms’ infestation prevalence, with EpiInfo 6 [3], and Mann and Whitney test was used to compare farms’ infestation abundance and intensity. All the comparisons were made at the threshold of 0.05. The relationship between birds’ age and parasite burden was studied with CurveExpert Release 1.4 [8].

## Results and discussion

A total of 329 *D. gallinae* were collected from 13 units out of 38, totalling 219,508 birds. There was no statistically significant difference between the infestation prevalence of egg-laying hen farms and that of reproductive hen farms (*p* > 0.05); this may be due to the small number of sampled farms. However, infestation intensity and abundance were statistically significantly higher in egg-laying hen farms than reproductive hen farms (Table 1). This can be explained by two factors: (i) the higher market value of reproductive poultry compared with egg-laying hens, leading to a higher level of health care provided by the farmers; (ii) the difference of housing management system, since mites’ hiding places were more frequent in egg-laying hens’ units. Birds’ infestation prevalence was by far higher in Italy; indeed, Cafiero et al. [1] estimated this prevalence to be 74%. There was no correlation between infestation intensity and room temperature (*R* ^2^ = 0.0001; *p* > 0.05). This can be explained by the presence of a room temperature range suitable for red mite survival in all units, which were air-conditioned, with the temperature varying between 29 and 32 °C. Indeed, Tucci et al. [14] showed that the optimum temperature for *D. gallinae* development was 30 °C.

**Table 1.** Epidemiological indicators of poultry red mite infestations.

Poultry unit type	Prevalence (% ± *SE*)	Intensity	Abundance
Egg laying hen	11/29 (37.93 ± 0.09)^a^	0.0031^a^	0.0016^a^
Reproductive poultry	2/10 (20.00 ± 0.126)^a^	0.0005^b^	0.0003^b^
Overall	13/39 (34.21 ± 0.075)	0.0028	0.0015

Different letters in each column correspond to the presence of statistical significance.

The treatment of egg-laying poultry with chemical acaricides is forbidden because they represent a risk to the consumers. That is why we strongly recommend the use of new crates for transporting birds, which is an excellent control for introduced birds and allows a good acaricide treatment during the cleaning time between batches. The relationship between poultry age (breeding duration) and infestation intensity was exponential, *y* = 78.41(1 − exp(−0.009*x*), showing a fast increase of mite population. A sensitive screening technique during the first period of breeding followed by a fast implementation of suitable control measures in infested units are requested to avoid an exponential increase of mite population. The epidemiological indicators of the present survey were underestimated, since the farmers sprayed acaricides during the cleaning period between batches and we placed the traps for only 24 h in a few places of the units. Hence, the presence of few parasites should be interpreted as a significant infestation requiring the implementation of control measures. Since the infestation prevalence of *D. gallinae* and its economic impact are high, and *D. gallinae* screening is cheap, animal health decision makers should recommend systematic screening in all poultry units during three periods: (i) during the cleaning time between batches; (ii) monthly, during the whole production cycle; and (iii) after any anti-mite treatment, allowing an evaluation of control efficiency. Positive units should be promptly treated to avoid the exponential increase of mite population and thus a decrease of control effectiveness and high financial production losses. Further studies are needed to estimate the sensitivities of the screening methods which, to our knowledge, lack sensitivity and need to be improved.
